# COVID-19 and Selenium Deficiency: a Systematic Review

**DOI:** 10.1007/s12011-021-02997-4

**Published:** 2021-11-05

**Authors:** Mohammad Fakhrolmobasheri, Sadegh Mazaheri-Tehrani, Marek Kieliszek, Mehrdad Zeinalian, Mehdi Abbasi, Fateme Karimi, Amir Mohamad Mozafari

**Affiliations:** 1 Isfahan Cardiovascular Research Center (Heart Failure Research Center), 81746-73461 Isfahan, Iran; 2 Child Growth and Development Research Center, Research Institute for Primordial Prevention of Non-Communicable Disease, Isfahan University of Medical Sciences, 81746-73461 Isfahan, Iran; 3 Department of Food Biotechnology and Microbiology, Institute of Food Sciences, Warsaw University of Life Sciences − SGGW, Nowoursynowska 159C, 02-776 Warsaw, Poland; 4 Department of Genetics and Molecular Biology, School of Medicine, Isfahan University of Medical Sciences, 81746-73461 Isfahan, Iran; 5 School of Medicine, Isfahan University of Medical Sciences, 81746-73461 Esfahan, Iran; 6 Medical Library and Information Sciences Department, Health Information Technology Research Center, School of Management and Medical Information Sciences, Isfahan University of Medical Sciences, 81746-73461 Isfahan, Iran

**Keywords:** Selenium, COVID-19, SARS-CoV-2, Oxidative stress, Micronutrients, Trace elements

## Abstract

Several studies have indicated that selenium deficiency may be detrimental in the context of various viral disorders, and in the case of COVID-19, several studies have reported heterogeneous results concerning the association of selenium deficiency with the severity of disease. To summarize the available data surrounding the association of body selenium levels with the outcomes of COVID-19, a systematic search was performed in the Medline database (PubMed), Scopus, Cochrane Library, Embase, and Web of Science using keywords including “SARS-CoV-2,” “COVID-19,” and “selenium,” Studies evaluating the association of COVID-19 with body selenium levels were included. Among 1,862 articles viewed in the database search, 10 articles were included after title, abstract, and full-text review. One study was further included after searching the literature again for any newly published articles. Out of 11 included studies, 10 studies measured serum selenium level, and one study investigated urinary selenium level. Three of 10 studies measured serum SELENOP level as well as selenium level. Glutathione peroxidase-3 level in serum was also assessed in one study. The reported outcomes were severity, mortality, and risk of COVID-19. Nine studies indicated that a lower serum selenium level is associated with worse outcomes. Two studies reported no significant association between serum selenium level and COVID-19. In one study, urinary selenium level was reported to be higher in severe and fatal cases compared to non-severe and recovered patients, respectively. In most cases, selenium deficiency was associated with worse outcomes, and selenium levels in COVID-19 patients were lower than in healthy individuals. Thus, it could be concluded that cautious selenium supplementation in COVID-19 patients may be helpful to prevent disease progression. However, randomized clinical trials are needed to confirm this.

## Introduction

Since January 2020, the world has faced a serious pandemic of coronavirus disease 2019 (COVID-19). The disease, which is caused by severe acute respiratory syndrome coronavirus 2 (SARS-COV-2), was first reported in the city of Wuhan in Hubei province of China and rapidly spread all over the world [1, 2]. The clinical course of COVID-19 can lead to a wide variety of complications including acute respiratory distress syndrome (ARDS), systemic inflammatory response syndrome (SIRS), and multiple organ dysfunction syndrome (MODS). Recent studies suggest that the disturbances in cellular redox states and severe inflammatory response accompanied by a massive cytokine storm may be key contributors in the progression of COVID-19 to a severe or fatal disease [3–5].

Trace elements are dietary components that are required in small amounts but play pivotal roles in the homeostasis of the immune system. They mostly act as catalysts in enzymatic reactions in the context of viral diseases. Investigations have indicated a powerful link between an imbalance in the levels of trace elements (e.g., iron, zinc, copper, selenium, and magnesium) and disease severity [6, 7].

Selenium is a trace element that is a constituent of the 21st amino acid, selenocysteine. There are 25 different known genes that code various proteins containing selenium (selenoproteins). Selenoproteins in play various functions in the human body, including redox reactions and cell signaling (e.g., glutathione peroxidase and thioredoxin reductase), activation and proliferation of immune cells (selenoprotein K), and selenium transport (selenoprotein P or SELENOP) [7–9]. It has been reported that selenium deficiency is associated with a higher risk of several chronic diseases with inflammatory pathogenesis, including cancer and cardiovascular disorders [8]. In viral disorders, selenium supplementation has indicated positive results [10]. This could be due to the anti-inflammatory, immune boosting, and antithrombotic effects of selenium [11]. Moreover, it has been proven that selenium deficiency is associated with a higher viral genome mutation rate in various viral infections caused by RNA viruses such as HIV, Ebola virus, Coxsackievirus, hantavirus, influenza virus, and SARS-CoV [12, 13].

Several studies have assessed the relationship between the level of body selenium and the incidence, severity, and mortality of COVID-19. Also, there some studies have investigated the correlation between the selenium content of soil and COVID-19 severity and prevalence [14]. However, although most of the studies have suggested that selenium deficiency is associated with worse outcomes in COVID-19, there seems to be a heterogeneity in the reported results [15, 16]. Although there are several review studies on trace elements and COVID-19, to our knowledge, there is no systematic study investigating specifically the association of selenium with outcomes of COVID-19. Thus, we planned to conduct a systematic review to better clarify the association between selenium status and COVID-19 and whether selenium supplementation could ameliorate the disease course in COVID-19 patients.

## Materials and Methods

### Search Strategy

This review was conducted according to the Preferred Reporting Items for Systematic Reviews and Meta-Analyses (PRISMA) statement [17]. A systematic search was performed in the Medline database (PubMed), Scopus, Cochrane Library, Embase, and Web of Science up to 27 June 2021 using the following search line: (“2019 novel coronavirus disease” OR “2019 novel coronavirus infection” OR “2019-nCoV disease” OR “2019-nCoV infection” OR COVID OR “COVID 19” OR “COVID 2019” OR “COVID-19” OR “COVID19” OR “SARS coronavirus 2 infection” OR “SARS-CoV-2 disease” OR “SARS-CoV-2 infection” OR “SARS-CoV2 disease” OR “SARS-CoV2 infection” OR “SARSCoV2 disease” OR “SARSCoV2 infection” OR “Wuhan coronavirus disease” OR “Wuhan coronavirus infection” OR “coronavirus disease 2019” OR “nCoV 2019 disease” OR “nCoV 2019 infection” OR “novel coronavirus 2019 disease” OR “novel coronavirus 2019 infection” OR “novel coronavirus disease 2019” OR “novel coronavirus infection 2019”) AND (selenium OR se OR selenicum). We reviewed and screened the papers based on title, abstract, and full-text review. Additionally, related review articles were checked to find undetected relevant studies. During the article writing process, a new update of each database was screened to consider any new published article suitable to include in the review process.

### Inclusion and Exclusion Criteria

All original English-language articles studying the relation between human body selenium level and COVID-19 were included. There was no restriction for gender, race, ethnicity, or publication date. In silico, in vivo, and in vitro investigations, animal studies, and duplicated publications were excluded.

### Data Extraction

The following information was extracted from included records: author name, publication date, study design, sample size, demographic features of sample, selenium level, and outcomes.

### Quality Assessment

The STROBE checklist was used for quality assessment [18]. This checklist is designed for cohort, case control, and cross-sectional studies. It consists of 22 items for different parts of an article, including title and abstract (1 item), introduction (2 items), material and method (9 items), result (5 items), discussion (4 items), and funding (1 item). Scores can vary from 0 to 22 points.

## Result

### Studies Included

**Fig. 1:**
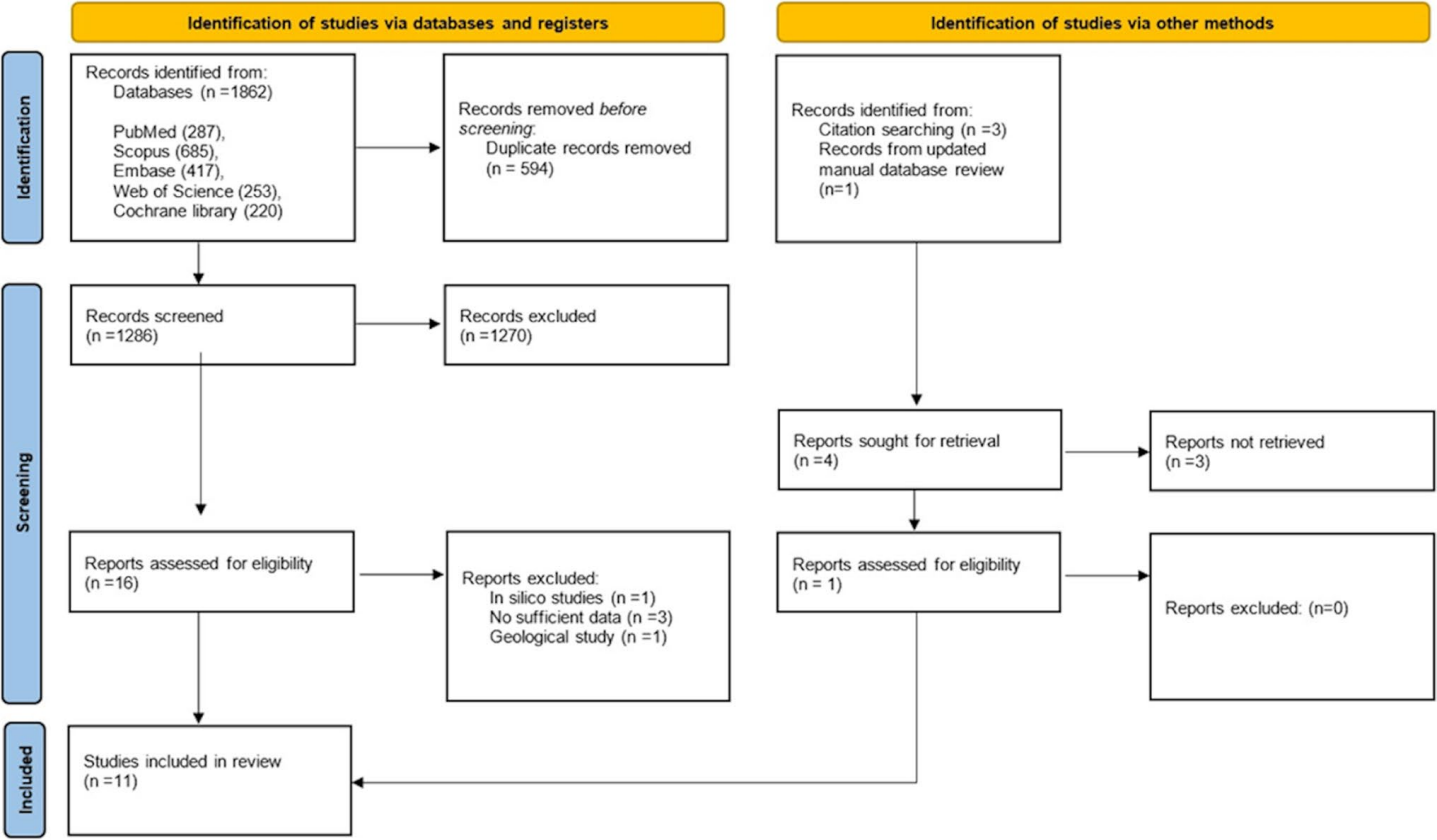
PRISMA flow diagram of the study selection

Figure 1 summarizes the process of study selection. In the initial search, 1,862 articles was found; 1,286 articles remained after the removal of duplicates. After title and abstract screening, 16 papers qualified for further assessments. The full texts of remaining articles were reviewed carefully by three independent reviewers. The study by Erol et al. [19] was included during the daily updated review of the database. Finally, based on the eligibility criteria, 11 studies were included in the systematic review.

### Features of Included Studies

All the included articles utilized observational design. Out of 11 included studies, 9 used cross-sectional analysis, one utilized retrospective analysis, and one was conducted in a prospective manner. Ten studies measured serum selenium level, while one study investigated urinary selenium level. Three of the 10 studies measured serum SELENOP level as well as selenium level. Glutathione peroxidase-3 level in serum was also assessed in one study.

Up to now, studies have assessed the effect of serum selenium status on the severity, mortality, and risk of COVID-19. Some of them surveyed more than one of these consequences. Three studies discussed the relationship between serum selenium level and severity of COVID-19, three papers investigated the relationship between serum selenium level and mortality of COVID-19, and four studies compared the selenium level between COVID-19 patients and healthy individuals or reference intervals. The quality of the included studies was assessed using the STROBE checklist. The lowest score was 16, and the highest was 20. Table 1 shows scores for each article.

**Table 1:** Qualification assessment of included articles

Variables		Alkattan et al. 2021 [15]	Heller et al. 2020 [20]	Im et al. 2020 [21]	Majeed et al. 2021 [16]	Moghaddam et al. 2020 [22]	Muhammad et al. 2021 [23]	Pincemail et al. 2021 [24]	Skalny et al. 2021 [25]	Erol et al. 2021 [19]	Zeng et al. 2021 [26]	Hackler et al. 2021 [27]
Title/abstract	Title/abstract	1	1	1	1	1	1	1	1	1	1	1
Introduction	Background	1	1	1	1	1	1	1	1	1	1	1
	Objectives	1	1	1	1	1	1	1	1	1	1	1
Method	Design	1	1	1	1	1	1	1	1	1	1	1
	Setting	1	1	1	1	1	1	1	1	1	1	1
	Participants	1	0	1	1	1	1	1	1	1	1	1
	Variables	0	1	1	1	1	1	1	1	1	1	1
	Measurement	1	1	1	1	1	1	1	1	1	1	1
	Bias	0	1	0	0	0	0	0	0	0	1	0
	Study size	0	0	0	0	0	0	0	0	0	0	0
	Quantitative variables	1	1	0	1	1	0	1	1	1	1	0
	Statistical methods	1	1	1	1	1	1	1	1	1	1	1
Result	Participants	1	1	1	1	1	1	1	1	1	1	1
	Descriptive data	1	1	1	1	1	1	1	1	1	1	1
	Outcome data	1	1	1	1	1	1	1	1	1	1	1
	Main results	1	1	1	1	1	1	1	1	1	1	1
	Other analyses	0	0	1	1	1	1	1	1	1	1	1
Discussion	Key results	0	1	1	0	1	1	1	1	1	1	1
	Limitations	1	1	1	0	1	1	1	0	0	1	0
	Interpretation	1	1	1	1	1	1	1	1	1	1	1
	Generalizability	1	0	0	0	0	1	1	0	1	0	0
Other	Funding	0	1	1	1	1	1	1	1	1	1	1
Total	Score out of 22	16	18	18	17	19	19	20	18	19	20	17

Overall, the 11 included studies cover 681 COVID-19 patients and 164 healthy individuals. The sample size of the included articles varies from 9 to 193. Investigations were conducted in different countries: three in Germany, one in Belgium, one in Russia, one in Turkey, one in Saudi Arabia, one in South Korea, one in India, one in Nigeria, and one in China. Table 2 summarizes the characteristics of the included articles.

**Table 2:** Summary of studies assessing selenium level in COVID-19 patients

Author	Date	Country	Design	Sample	Participants	Age^1^ (year)	Male (%)	Selenium^1^ (ng/mL)	Se normal range	Outcome
Alkattan et al. 2021 [15]	January 2021	Saudi Arabia	Cross-sectional	Blood	80 COVID-19 patients (severe = 35, non-severe = 45)	51.54	64.9%	Severe = 162, non-severe = 134, all cases = 138	70–150	Se level was significantly more in severe cases (*p* < 0.0001)
Heller et al. 2020 [20]	October 2020	Germany	Cross-sectional	Blood	35 COVID-19 patients (discharge = 29, death = 6)	77 ± 41.48	46%	N/A	N/A	Se and SELENOP was significantly increase in discharges during hospitalization unlike deaths
Im et al. 2020 [21]	August 2020	South Korea	Cross-sectional	Blood	50 hospitalized COVID-19 patients	57.5 ± 24.81	58%	98.3 ± 12.81	More than 95	Se deficiencies increases severity and mortality
Majeed et al. 2021 [16]	November 2020	India	Cross-sectional	Blood	30 COVID-19 & 30 healthy individuals	37	63.3%	Control = 79.09 ± 10.9, patients = 69.26 ± 8.78	70–150	Se level was significantly lower in patients (*p* < 0.0003)
					14 healthy & 24 COVID-19 individuals	N/A	100%	Control = 79.4 ± 9.2, patients = 68.4 ± 8.9	70–150	Se level was significantly lower in patients (*p* < 0.0012)
					16 healthy & 6 COVID-19 individuals	N/A	0%	Control = 79.5 ± 12.5, patients = 74.9 ± 5.9	70–150	No significant difference
Moghaddam et al. 2020 [22]	July 2020	Germany	Cross-sectional	Blood	33 COVID-19 patients (discharge = 27, death = 6)	77 ± 41.48	42%	Selenium (discharge = 53.3 ± 16.2, death = 40.8 ± 8.1)/SELENOP [mg/L] (discharge = 3.3 ± 1.3, death = 2.1 ± 0.9)/GPx3[U/L] (discharge = 251.6 ± 69.6, death = 224.8 ± 30.3)	45.5–131.6	Se and SELENOP level was significantly lower in deaths (*p* < 0.001) & lower in patients comparing to reference data (*p* < 0.001) GPx3 was significantly lower in deaths (*p* < 0.001)
Muhammad et al. 2021 [23]	January 2021	Nigeria	Cross-sectional	Blood	50 COVID-19 & 21 healthy individuals	41.43	64.8%	Control = 29.1 ± 1.9 ng/dL, patients = 25.3 ± 2.4 ng/dL	N/A	Se level was significantly lower in patients (*p* < 0.001 (
Pincemail et al. 2021 [24]	February 2021	Belgium	Cross-sectional	Blood	9 COVID-19 (6 long stayers & 3 short stayers)	64 ± 13.33	88.8%	Long stayers = 97 ± 7.75, short stayers = 51 ± 9.75, all cases = 74 ± 11	73–110	Se level was significantly lower in short stayers (*p* = 0.023),
Skalny et al. 2021 [25]	April 2021	Russia	Cohort (prospective)	Blood	43 healthy & 150 COVID-19 (mild = 50, moderate = 50, severe = 50)	56.2	56%	Control = 102 ± 16, mild = 93 ± 20, moderate = 90 ± 22, severe = 87 ± 31	N/A	Se level was significantly lower in moderate (*p* = 0.047) and severe (*p* < 0.001 ( patients compared to control
Erol et al. 2021 [19]	May 2021	Turkey	Cross-sectional	Blood	26 healthy & 24 COVID-19 pregnant women in the 1st trimester	27.31	0%	Control = 44.59 ± 8.4, patients = 46.52 ± 8.17	N/A	No significant difference
					22 healthy & 26 COVID-19 pregnant women in 2nd Trimester	28.97	0%	Control = 46.15 ± 8.15, patients = 36.03 ± 9.86	N/A	Se level was significantly lower in patients (*p* < 0.001)
					22 healthy & 21 COVID-19 pregnant women in the 3rd trimester	27.59	0%	Control = 36.15 ± 6.25, patients = 27.01 ± 7.82	N/A	Se level was significantly lower in patients (*p* < 0.01 )
Zeng et al. 2021 [26]	December 2020	China	Cohort (retrospective)	Urine	138 hospitalized COVID-19 (severe = 68, non-severe = 70) * creatinine-adjusted	61.5 ± 9.6	57.2%	Severe = 45.63 ± 29.51 μg/g, non-severe = 27.65 ± 9.97 μg/g	15.86–38.13 μg/g	Se urinary level was significantly higher in severe cases (*p* < 0.001)
					138 hospitalized COVID-19 (severe = 68, non-severe = 70) * creatinine non-adjusted	61.5 ± 9.6	57.2%	Severe = 20.27 ± 16.15 μg/L, non-severe = 25.55 ± 13.78 μg/L	10.46–82.71 μg/L	Se urinary level was significantly lower in severe cases (*p* = 0.024)
					138 hospitalized COVID-19 (discharge = 117, death = 21) * creatinine-adjusted	N/A	N/A	Discharge = 40.56 ± 22.89 μg/g, death = 66.75 ± 56.84 μg/g	15.86–38.13 μg/g	Se urinary level was significantly higher in deaths (*p* < 0.001)
					138 hospitalized COVID-19 (discharge = 117, death = 21) * creatinine non-adjusted	N/A	N/A	Discharge = 19.95 ± 16.4 μg/L, death = 25.5 ± 18.36 μg/L	10.46–82.71 μg/g	No significant difference (*p* = 0.543)
Hackler et al. 2021 [27]	May 2021	Germany	Cross-sectional	Blood	35 hospitalized COVID-19 (discharge = 28, death = 7)	77 ± 41.48	42.9%	N/A	N/A	Se and SELENOP was significantly increase in discharges during hospitalization unlike deaths

Abbreviations: *Se*, selenium; *SELENOP*, selenoprotein P; *GPx3*, glutathione peroxidase 3

^1^Values are mean ± SD

### Serum Selenium Level in COVID-19 Patients vs Healthy Individuals

Majeed et al. [16] conducted a cross-sectional study of 30 healthy individuals (as a control) and 30 COVID-19 patients in India. The results showed that patients had significantly lower serum selenium levels compared to healthy participants. Further analysis was performed among both genders separately. In males, the results were the same, but in females, no significant difference found between serum selenium status in controls and cases. Moreover, despite significant difference in ages within controls and cases, further analysis showed that variation in age did not contribute to a difference in serum selenium level.

Another cross-sectional study [23] on 50 COVID-19 patients and 21 apparently healthy individuals in Nigeria found a significant decrease in circulating selenium among patients compared to controls.

An observational study [19] in Turkey on 141 pregnant women (71 COVID-19 and 70 healthy individuals) in different trimesters reported lower selenium levels in the second and third trimesters in patients compared to healthy individuals.

A cross-sectional study [24] in Belgium was carried out to find out the relationship between the duration of COVID-19 patients’ hospitalizations in intensive care unit (ICU) and trace elements status. Analysis revealed that short stayers (7–11 days) had a far lower level of selenium than reference data, but long stayers (38–43 days) had a normal level of selenium. There was a significant difference in circulating selenium between the 2 groups.

### Serum Selenium Level and Severity of COVID-19

A cross-sectional study [15] was conducted on 80 COVID-19 patients in Saudi Arabia. The researchers divided patients into 2 groups (severe = 35, non-severe = 45) based on the diagnostic and treatment guidelines for SARS-CoV-2 issued by the Chinese National Health Committee [28]. Their results showed that serum selenium level was significantly higher in severe patients compared to non-severe patients. They suggested that elevated serum selenium level (selenosis) may lead to dysfunction of pathways related to the endoplasmic reticulum (ER) stress that increases pro-inflammatory prostaglandin formation.

Another prospective observational study, conducted by Skalny et al. [25] in Russia, measured serum selenium status in 150 confirmed COVID-19 and 43 healthy participants. Based on the guidelines of the Russian Ministry of Healthcare, patients were categorized into three groups (50 mild, 50 moderate, and 50 severe cases). They reported a significant decrease in serum selenium levels among severe and moderate cases compared to controls, but no significant was difference found between mild cases and controls. Analysis also indicated that circulating selenium level correlated directly with oxygen saturation but inversely with lung damage, CT grade, CRP levels, and fever. A cross-sectional study [21] of 50 hospitalized COVID-19 patients in South Korea showed the same results. In patients with severe disease, selenium deficiency was observed more frequent (in 42% of patients).

### Serum Selenium Level and Mortality of COVID-19

Analysis of a cross-sectional study [20] in Germany of 35 COVID-19 patients (171 serum samples) showed that circulating selenium and SELENOP concentrations increased in the discharge group (*n* = 29) during hospitalization and unlike deaths (*n* = 6), and generally the amount of selenium and SELENOP in serum was higher in discharged patients compared to deaths. These authors also investigated the circulating level of selenium and SELENOP in 35 patients (173 serum samples, discharges = 28, deaths = 7) in another study, and the results go along with each other [27]. Another study [22] conducted by the same research team on 33 COVID-19 patients suggested remarkably lower levels of serum selenium, SELENOP, and glutathione peroxidase-3 in deaths comparing to discharges. According to their results, COVID-19 patients showed a notable deficit in total selenium and SELENOP concentrations in comparison with reference data from a European survey on 1,915 adults.

### Urinary Selenium Level and Severity and Mortality of COVID-19

One study run by Zeng et al. [26] in China assessed urinary trace elements of 138 COVID-19 patients. Patients were divided into 2 groups (severe = 68, non-severe = 70) according to the Guidelines of the Diagnosis and Treatment of New Coronavirus Pneumonia published by the National Health Commission of China. The results showed that urinary selenium level was significantly lower in severe cases compared to non-severe ones. However, when the results were adjusted by the urinary creatinine, severe patients showed a significantly higher amount of selenium excretion. The researchers reported no significant difference between urinary selenium level in deceased and recovered groups, but when adjusted for urinary creatinine, a higher level of urinary selenium in deaths (*n* = 21) compared to discharges (*n* = 117) was observed.

## Discussion

A primary search in the Medline database using the keywords COVID-19 and selenium results in more than 100 titles, of which around 40 titles remain using the filter for review articles. However, almost none of these published review articles aimed to systematically review the available clinical studies surrounding the association of selenium with COVID-19 outcomes. Thus, in this systematic review, we aimed to provide stronger evidence about the association of body selenium status with COVID-19 and its outcomes.

Selenium is one of the essential trace elements in the human body, playing a pivotal role in modulating the function of the immune system, maintaining redox homeostasis, and diminishing inflammatory cytokine cascade [29]. Previously, we suggested several molecular mechanisms for the antiviral and anti-inflammatory effects of selenium supplementation, whereas both the immune boosting and the antioxidant effects of selenium are demonstrated in clinical studies [3]. To date, there are several studies suggesting a relationship between selenium deficiency and chronic inflammatory diseases such as cardiovascular, subfertility, cancer, and viral infections [13, 30, 31].

There are several dietary sources of this metalloid, such as tuna, sardines, shellfish, chicken, eggs, nuts, and cereals [32]. Plant products contain inorganic forms of selenium, including selenates (IV) and (VI). In the human body, these are converted to the organic forms, mainly selenomethionine and selenocysteine [33]. Recent geological studies indicated that the nutritional status of selenium depends on the selenium content of soil. Regarding the uneven distribution on Earth, large differences in selenium content are observed between different regions [34–36].

The story of Keshan disease, a great example of the effect of regional soil selenium status on the outcomes of a viral disease (Coxsackie B virus)-induced cardiomyopathy, will now be joined by the many experiences in the context of COVID-19 and selenium [37]. As an example, agricultural products in Finland, unlike in Sweden, are supplemented with selenium. Although Sweden and Finland have equal access to healthcare, up to July 2020, deaths due to COVID-19 in Sweden are about ten times higher. This huge difference might be because of a different selenium status between the mentioned countries [38]. On the other hand, a study on the incidence rate of COVID-19 in different cities of Hubei suggested a relationship between selenium soil content and the incidence of disease [14]. Another geological study on 14,045 COVID-19 cases from 147 cities of China showed that regional selenium deficiency might be associated with the fatality of COVID-19 patients [39]. Zhang et al. [40] reported a significant positive correlation between the COVID-19 cure rate and hair selenium concentration, as a validated measure of selenium intake [41], in 17 cities in China (*R*^2^ = 0.72, *F* test *p* < 0.0001). These investigations demonstrate the importance of selenium in protection against COVID-19.

In addition to the geological studies, clinical studies have also evaluated the hypothesis around the relation between COVID-19 and selenium deficiency. As demonstrated in the Results section, the data extracted from the included articles indicated that 1. lower serum selenium was observed in fatal cases compared to recovered patients; 2. serum selenium status was lower in severe COVID-19 patients versus the mild–moderate patients; and 3. comparing COVID-19 patients to healthy individuals, serum selenium levels in COVID-19 patients was lower. It was also reported that serum selenium and SELENOP level increase gradually in patients during the period of recovery.

Although most studies indicate that selenium deficiency is associated with worse outcomes in COVID-19 patients, Alkattan et al. [15] indicated a higher level of selenium in severe patients in comparison with the non-severe cases. This contradiction may be explained by the study limitations mentioned by the authors, including a lower sample size or lower confidence level (80%), and also the fact that in both severe and non-severe groups, the serum selenium levels were within normal limits and the samples were collected in a limited period of time (24 h). Erol et al. [19] discussed serum selenium levels in samples from pregnant women. Selenium deficiencies in pregnant women were observed to be associated with higher risk of preterm delivery and low birth weight [42]. Maternal selenium status significantly decreases during pregnancy due to an increased need for selenium [43]. Their results suggest a decrease in maternal selenium level during the second and third trimester in COVID-19 patients compared to healthy participants. Their results not only demonstrate the importance of adequate selenium intake in this special group of patients but also may pose the question whether the particular changes in immune system during the pregnancy is associated with body selenium status and the outcomes of COVID-19.

There are large differences in the reported levels of selenium in different studies, particularly the study by Muhammed et al. [23], which reported serum selenium levels of 29 ng/dL in healthy individuals and 25 ng/dL in COVID-19-infected patients. This could be explained by the considerably low levels of selenium in soil in Nigeria [44].

Up to now, there is just one study that has measured the level of urinary trace elements in COVID-19 patients. Zeng et al. [26] reported that the creatinine-adjusted urinary selenium level in severe cases was higher compared to non-severe cases. Also, it was indicated to be higher in the deceased group compared to recovered patients. This result raises questions surrounding the renal function in severe COVID-19: whether renal injury contributes to the greater selenium loss or whether the selenium levels in severe COVID-19 patients is higher compared to the mild/moderate patients. According to the studies evaluating kidney function in severe COVID-19 patients, it would be more reasonable to assume impaired kidney function as a reason for greater urinary selenium loss [45, 46].

Recent studies have demonstrated the efficiency of selenium supplementation in viral infections such as polio, influenza, HIV, hantavirus, and SARS-CoV [14, 47, 48]. The decreased level of selenium among COVID-19 patients and its association with severity and mortality of disease may suggest the application of selenium supplementation for COVID-19 cases, but it has not been proven yet in large-scale clinical trials [4, 11, 49]. There is a narrow range between an appropriate and a toxic amount of this metalloid, so selenium supplements should be taken with caution [50]. The recommended amount of daily selenium intake is 1 μg per kg of body weight. According to D-A-CH reference, men are recommended to consume 70 μg of selenium per day, and women are recommended to take 60 μg per day. The estimated values for pregnant and lactating women are 60 and 75 μg per day, respectively [35, 51]. The required serum selenium level is reported to be between 130 and 150 ng/mL [8]. However, the US Food and Drug Administration observed no adverse effect level for a serum selenium concentration of 1000 ng/mL [52]. Excess amounts of selenium may cause many complications including hair loss, fatigue, gastrointestinal disorders, and increasing risk for type 2 diabetes [8]. So, monitoring the level of selenium is very important. Nano-selenium is a supplement that has shown lower toxicity and more bioavailability compared to routine selenium supplement. Studies have demonstrated its utility for different complications including cancers [53] and Huntington’s disease [54]; also He et al. [55] discuss its efficiency against COVID-19.

Additionally, a considerable aspect of selenium supplementation is associated with mutations in RNA viruses. Prior studies on influenza and Coxsackie viruses have demonstrated that in selenium-depleted hosts, there is a much greater chance for viral genome mutation [13]. This goes along with the recent dramatic health threats caused by new variants of SARS-CoV-2 [56]. Thus, it might be considered that keeping general population from becoming selenium deficient may prevent SARS-CoV-2 from further dangerous mutations.

In conclusion, most of the examined COVID-19 patients indicated a low selenium level. Selenium deficiency might be considered as an indicator for the severity, mortality, and overall risk of COVID-19. Regarding the mentioned facts, selenium might be useful as a supplement for COVID-19 patients, but further clinical trials are needed to clarify its efficiency.

## Data Availability

All data are available with request to the corresponding author.
